# The Clinical Ultrasonography Elective in Clerkship (CUSEC): A pilot elective for senior clerkship students at the University of Saskatchewan

**DOI:** 10.36834/cmej.61810

**Published:** 2020-03-16

**Authors:** Paul Olszynski, Mackenzie Russell, Adam Neufeld, Greg Malin

**Affiliations:** 1 College of Medicine, University of Saskatchewan, Saskatchewan, Canada; 2 Department of Emergency Medicine, Royal University Hospital, Saskatchewan, Canada; 3 Department of Academic Family Medicine, Royal University Hospital, Saskatchewan, Canada

## Abstract

We created a clinical ultrasound (CUS) elective in clerkship, which gave medical students the opportunity to enhance their knowledge and technical skills while refining their CUS-related clinical decision making. This elective uniquely allowed medical students to integrate their CUS knowledge and skills into real patient care within the clinical environment (discipline) of their choice. As such, beyond supporting increasing technical competence, students learned to advocate for appropriate use of CUS, an important skill for trainees to develop.

## Déclaration des répercussions

Nous avons créé un stage d’externat optionnel enéchographie ciblée, laquelle offre aux étudiants en médecine l’occasion d’améliorer leurs connaissances et habiletéstechniques tout en raffinant leurs prises de décisions cliniques liées à l’échographie ciblée. Ce stage optionnel offre une occasion unique aux étudiants en médecine d’intégrer leurs connaissances et habiletésen échographie cibléeàdes soins réels aux patients dans un environnement clinique (discipline) de leur choix. Ainsi, au-delà d’un soutien au développement d’une compétence technique,les étudiants ont appris à préconiser une utilisation appropriée de l’échographie ciblée, une habiletéimportante à développer pour les apprenants.

## Introduction

Teaching ultrasonography in medical schoolisbecoming increasingly common,^1^^,^^2^both as a learning tool^3^^,^^4^anda clinical skill in itself.^5^ All medical students in the undergraduate medical education program at the University of Saskatchewan receive ultrasound training. During pre-clerkship years 1 and 2, students learn through lectures and supervised hands-on scanning during their Anatomy and Clinical Skills courses. However, opportunities to further develop clinical ultrasonography skills during clinical rotations in clerkship remain sporadic and preceptor dependent. Given that medical students do not routinely encounter CUS during core clinical rotations,^6^^,^^7^ several medical schools have developed CUS electives focused on introducing or further developing students’ image generation and interpretation skills. There are no reports in the literature on CUS electives that also provide students with robust opportunities to integrate their CUS skills into patient care within the clinical learning environment. Such an experience would go beyond skill development and support the learner in making clinical decisions about appropriate use of CUS, a key skill to develop that supports patient care.

## Innovation

A clinical ultrasonography elective in clerkship (CUSEC) was developed to provide senior clerkship students with an opportunity to advance and integrate their skills into real patientcare. Twelve medical students participated in the 2-week CUSEC. Week 1 consisted of CUS teaching, image interpretation sessions, and supervised scanning on standardized patients. During Week 2, students joined a clinical rotation of their choice, where they integrated CUS into regular clinical practice with patients. Week 1 assessment included multiple-choice questions (MCQs) testing their image interpretation and clinical integration knowledge, followed by a practical examination,^8^ testing their image generation and communication skills. In Week 2, clinical rotation supervisors completed clinical assessment forms, describing the appropriateness and reliability of the students’ CUS skills in the context of everyday patient care.

Students provided evaluations of their CUSEC learning experiences. The University of Saskatchewan Research Ethics Board considered this research to be a “program evaluation,” and therefore exempt from ethical review. All students enrolled in CUSEC provided informed consent prior to participating in the CUSEC survey. Participants were advised that their test scores may be used for research purposes but would remain anonymous, and that their confidentiality would be maintained.

## Outcomes

**Figure 1:**
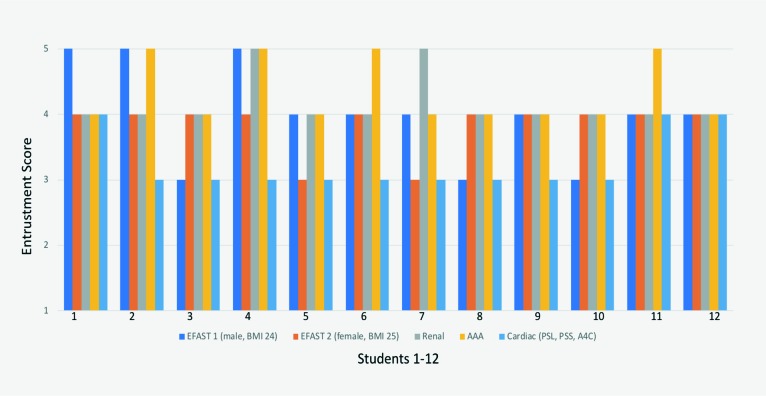
Practical exam entrustment scores for N = 12 students on Focused Assessment with Sonography in Trauma (FAST) scans (M = 4.0, SD = 0.74 and M = 3.8, SD = 0.39), Renal (M = 4.2, SD = 0.39), AAA (M = 4.33, SD = 0.49) and cardiac scans (M = 3.25, SD = 0.45) (1 = could not perform, 2 = lots of supervision, 3 = minimal prompting, 4=independent but lacking in efficiency, 5 = proficient; M = mean, SD = standard deviation)

Student MCQ marks on the Week 1 exam ranged from 80-95%. As seen in Figure 1, all students generated adequateCUSviews with,atmost,minimal prompting, which translated into specific entrustment scores, for assessment.Several studentswereable to generate excellent images with no guidance from the examiner at all.

Clinically, students met or exceeded expectations in the safe integration of clinical ultrasonography. Medical students reported CUSEC to be relevant, motivating, and beneficial for learning bed-side patient care.

### Next steps

We provide an approach to the implementation of CUS in clerkship years that builds on clerkship learning goals and supports CUS integration to clinical contexts. CUSEC students demonstrated high quality technique and knowledge. Students were very satisfied with the elective, describing it as valuable with content analysis identifying themes of autonomy, mastery, and relevance. We will continue to offer this elective in clinical ultrasonography and intend to explore its effectiveness in more detail.
